# Pharmacokinetic/Pharmacodynamic Analysis of High-Dose Daptomycin in Combination with Continuous Infusion Ceftobiprole in a Case Series of Documented Staphylococcal Bacteremia or Endocarditis: Is There Any Room for TDM-Guided Dosing Reduction?

**DOI:** 10.3390/antibiotics15030315

**Published:** 2026-03-19

**Authors:** Pier Giorgio Cojutti, Renato Pascale, Andrea Grechi, Simone Ambretti, Pierluigi Viale, Federico Pea

**Affiliations:** 1 Department of Medical and Surgical Sciences, Alma Mater Studiorum, University of Bologna, 40138 Bologna, Italy; piergiorgio.cojutti@unibo.it (P.G.C.); renato.pascale2@unibo.it (R.P.); simone.ambretti3@unibo.it (S.A.); pierluigi.viale@unibo.it (P.V.); 2 Clinical Pharmacology Unit, Department of Integrated Infectious Risk Management, IRCCS, Azienda Ospedaliero-Universitaria di Bologna, 40138 Bologna, Italy; 3 Infectious Diseases Unit, Department of Integrated Infectious Risk Management, IRCCS, Azienda Ospedaliero-Universitaria di Bologna, 40138 Bologna, Italy; andrea.grechi3@unibo.it; 4 Microbiology Unit, Department of Integrated Infectious Risk Management, IRCCS, Azienda Ospedaliero-Universitaria di Bologna, 40138 Bologna, Italy

**Keywords:** daptomycin, continuous infusion ceftobiprole, bacteremia, endocarditis, PK/PD target attainment, model-informed precision dosing (MIPD)

## Abstract

Background: Staphylococcal bloodstream infections (BSIs) and infective endocarditis (IE) are associated with high morbidity and mortality. Among the different antimicrobial combination strategies proposed to enhance antibacterial activity, the association of daptomycin and ceftobiprole may be valuable. The aim of this study was to assess the PK/PD target attainment, safety, and clinical outcomes of such combination therapy for BSI and IE treatment. Methods: This retrospective monocentric study included adult patients with targeted treatment of staphylococcal BSI or IE with daptomycin plus continuous infusion (CI) ceftobiprole. Therapeutic drug monitoring (TDM) was performed for both agents, including Bayesian estimation of daptomycin 24 h area under the concentration–time curve (AUC_24h_). PK/PD targets were defined as daptomycin AUC_24h_/MIC ≥ 666 and ≥1081, and ceftobiprole steady-state concentration/MIC ≥ 4. Dose adjustments, safety, microbiological response, and clinical outcomes were assessed. Results: Twenty-three patients (11 BSI and 12 IE) were included. Methicillin-resistant *Staphylococci* were identified in 91.3% of cases. At first TDM assessment, daptomycin PK/PD targets were achieved in all patients, while ceftobiprole targets were achieved in 91.6% of BSI cases and in all IE cases. PK-/PD-guided dose de-escalation was frequently feasible. Clinical cure was observed in 77.8% of evaluable patients with BSI and in 91.7% with IE. Creatine phosphokinase elevations occurred in two patients, while hyper-eosinophilia was observed in 69.6% and was manageable with monitoring. Conclusions: Targeted therapy with daptomycin plus CI ceftobiprole achieved high PK/PD target attainment and favorable clinical outcomes in staphylococcal BSI and IE. TDM and model-informed precision dosing may enable dose optimization and may improve the balance between efficacy and safety. Multicenter studies are warranted.

## 1. Introduction

*Staphylococcus aureus* bacteremia is a major cause of morbidity and mortality worldwide, accounting for approximately 20–30% of all bloodstream infections (BSIs) in high-income countries [1,2]. Among *S. aureus* isolates, methicillin-resistant *S. aureus* (MRSA) accounts for 25–50% of strains in healthcare-associated settings, depending on different geographic areas [2,3]. BSIs caused by MRSA are associated with significantly worse outcomes compared to those caused by methicillin-susceptible strains, particularly among patients having comorbidities or delayed antimicrobial treatment, with mortality rates ranging between 20 and 40% [3,4].

Infective endocarditis (IE) is one of the most severe complications of *S. aureus* BSI, and may occur in as much as 10–25% of cases [5,6]. Overall, MRSA may account for approximately 20–35% of *S. aureus* IE and is associated with high mortality, often reported between 30 and 50%, especially when left-sided [1,7].

Also, coagulase-negative staphylococci (CoNS) may be major pathogens involved in healthcare-associated BSI and IE. Among these, *Staphylococcus epidermidis* may be responsible for approximately 20–30% of healthcare-associated BSI, and 70–80% of the clinical isolates are methicillin-resistant (MRSE) [8,9]. MRSE is often the etiological agent of prosthetic valve endocarditis, accounting for 20–30% of cases, with mortality rates of 15–30%, largely driven by biofilm formation and device-associated infection [5,9].

Current guidelines recommend vancomycin or daptomycin as first-line choice for treating MRSA/MRSE BSI with duration of at least 2 weeks in uncomplicated cases or 4–6 weeks in complicated, high-risk, or metastatic infections [10]. Likewise, the 2023 ESC Guideline recommends the same agents for treating MRSA/MRSE IE, but treatment duration should be guided by the underlying site of infection, namely 2–6 weeks in native-valve and ≥6 weeks in prosthetic-valve IE, respectively [11].

In recent years, the use of daptomycin is ever-growing in both of these settings compared to that of vancomycin. This is mainly because the vancomycin exposure needed for appropriately dealing with strains having borderline susceptibility to vancomycin (MIC ≥ 1 mg/L) may be associated with a high nephrotoxicity risk. Additionally, although daptomycin efficacy was shown to be at least comparable to that of vancomycin in several clinical studies [12,13], it is noteworthy that in two cohort studies of BSI caused by MRSA strains having borderline susceptibility to vancomycin, daptomycin resulted superior to vancomycin in both clinical outcome and survival rates [14,15]. Nowadays, the use of a high dose of daptomycin of 8–10 mg/kg/daily has become current practice both in empirical and targeted treatment, especially for complicated MRSA infections. This approach is considered effective in minimizing the risk of selecting daptomycin-tolerant or even resistant strains [16]. However, the use of daptomycin monotherapy for MRSA BSI is nowadays questioned and debated, especially in patients having persistent bacteremia or deep-seated/endovascular foci of infection [17]. Despite being standard of care, daptomycin monotherapy is still affected by quite high rates of both mortality (20–30%) [3,4,18] and microbiologic failure (10–16%) [19].

Interestingly, combining daptomycin with a fifth generation anti-MRSA cephalosporin, namely ceftobiprole or ceftaroline, might reveal effectiveness in this setting [20]. A phase 3, double-blind, double-dummy, noninferiority trial, carried out in adults with complicated *S. aureus* BSI, showed that ceftobiprole at a dose of 500 mg intravenously every 6 h for 8 days and every 8 h thereafter was non-inferior to daptomycin at a dose of 6 to 10 mg/kg daily [21]. Additionally, several in vitro studies showed that daptomycin and ceftobiprole may act synergically against MRSA [22,23,24]. This could be due to the fact that ceftobiprole may increase the bacterial cell-membrane daptomycin binding by decreasing the positive surface charge. A first retrospective clinical experience carried out among 11 cases receiving daptomycin plus ceftobiprole as rescue therapy of staphylococcal IE seemed promising [25]. Also, some single cases were reported [26,27]. Increasing evidence supports the use of continuous infusion as a strategy for maximizing the PK/PD target attainment of beta-lactams [23,28], some of which also concerned ceftobiprole [24,29].

Based on these assumptions, at our center in January 2024 we started treating cases of severe documented staphylococcal BSI and/or IE with daptomycin in combination with CI ceftobiprole [30] guided by therapeutic drug monitoring (TDM). CI is the best administration mode for maximizing the likelihood of PK/PD target attainment with a beta-lactam like ceftobiprole under the same daily dose [29].

The aim of this study was to perform a PK/PD analysis of high-dose daptomycin in combination with CI ceftobiprole in a prospective case series of documented staphylococcal BSI and/or IE receiving TDM-guided treatment.

## 2. Results

### 2.1. Patient Population

Twenty-three patients were included, namely 11 with BSI (11/23, 47.8%) and 12 with IE (12/23, 52.5%). Demographic and clinical characteristics are summarized in Table 1.

Median (IQR) age was 72 (61–82) years. Fourteen patients (14/23, 60.9%) were male, and the median (IQR) Charlson Comorbidity Index score was 6 (4–8). Median (IQR) body weight and estimated glomerular filtration rate (eGFR) were 70 kg (60–81 kg) and 69 mL/min/1.73 m^2^ (47–87 mL/min/1.73 m^2^), respectively. Most patients were admitted to the medical wards (13/23, 56.5%), followed by the intensive care units (7/23, 30.4%).

Among patients with BSI, the source of infection was primary in 5 cases (5/11, 45.5%), and secondary in the other 6 [2 cases each of pneumonia (18.2%), central venous catheter-related infection (18.2%) and deep abscesses (18.2%)]. Among patients with IE, the site of infection was a prosthetic valve in 8 cases (8/12, 66.7%), and a native valve in the remaining (4/12, 33.3%). At time of infection diagnosis, 2 patients (2/23, 8.7%) presented with septic shock. Overall, the median (IQR) SOFA score at diagnosis was 4 (2–6).

MRSA accounted for most of the clinical isolates (17/23, 73.9%), followed by MRSE (4/23, 17.4%) and MSSA (2/23, 8.7%). Daptomycin MIC value was ≤0.25 mg/L for all of the clinical isolates, whereas the median (IQR) ceftobiprole MIC was 0.5 mg/L (0.25–0.5 mg/L), and a maximum value of 1 mg/L was observed for two isolates.

### 2.2. Pharmacokinetic/Pharmacodynamic Analysis and Safety Considerations

The median (IQR) starting doses of daptomycin administered in the two groups were quite similar [8.9 (8.3–9.4) mg/kg daily in patients with BSI and 8.8 (6.9–9.9) mg/kg daily in those with IE]. At first daptomycin TDM assessment, median (min-max) AUC_24h_ was 1202.0 (584.0–1431.0) mg∙h/L in patients with BSI and 982.2 (425.0–1808.2) mg∙h/L in those with IE, respectively.

At the beginning of treatment, while receiving standard doses of daptomycin, all of the patients exceeded, frequently largely, both of the PK/PD targets of daptomycin efficacy [median (min–max) AUC_24h_/MIC ≥ 4808 (≥1356–≥5724) in patients with BSI and ≥3928.6 (≥1700–≥7211.2) in patients with IE].

**Figure 1:**
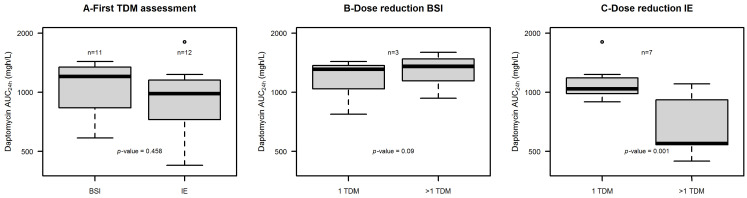
Box and whisker plot of daptomycin AUC_24h_ (mg·h/L). Panel (**A**) shows AUC_24h_ distribution at first TDM assessments in patients with BSI (*n* = 11) vs. patients with IE (*n* = 12). Panel (**B**) shows AUC_24h_ distribution at first TDM assessment vs. after dose reduction in patients with BSI with multiple TDM assessments and dose reduction (*n* = 3). Panel (**C**) shows AUC_24h_ distribution at first TDM assessment vs. after dose reduction in patients with IE with multiple TDM assessments and dose reduction (*n* = 7). The box plot represents the median with 25th–75th percentiles and the whiskers identify the range of normal data. Whiskers are calculated as 25th percentile − (1.5 × IQR) or 75th percentile + (1.5 × IQR), where IQR is the interquartile range. Dots are outliers.

Importantly, at first TDM assessment the daptomycin AUC_24h_ values were above the potential toxicity threshold of 939 mg∙h/L in more than half cases [54.5% of patients having BSI (6/11) and in 58.3% of those having IE (7/12)]. Consequently, in those patients undergoing subsequent TDM reassessments [5/11 of those with BSI (45.5%) and 9/12 of those with IE (75%)], daptomycin doses were reduced in most cases [60% of patients with BSI (3/5) and in 77.8% (7/9) of those with IE]. After dosing reduction, the median daptomycin AUC_24h_ values did not decrease in patients with BSI because of rapid worsening of renal function in all cases (1309 vs. 1354 mg∙h/L, *p* = 0.685). Conversely, in patients with IE the median daptomycin AUC_24h_ declined significantly (1039 vs. 547 mg∙h/L, *p* = 0.001). The distribution of daptomycin AUC_24h_ at first TDM assessment and after dose reduction for those patients having multiple TDM assessment in both groups is reported in Figure 1.

Importantly, both of the PK/PD targets of efficacy were still attained in the two groups [median (min-max) daptomycin AUC_24h_/MIC ≥5872 (≥3725.2–≥6476.4) in patients with BSI and ≥2900.2 (≥1784–≥5620) in patients with IE]. The attainment of the PK/PD targets for daptomycin at first TDM assessment and after dose reduction for those patients having multiple TDM assessment in both groups is reported in Table 2.

Likewise, the median (IQR) starting doses of CI ceftobiprole administered in the two groups were almost equal [1500 (1000–1500) mg/daily in patients with BSI and 1500 (875–1500) mg/daily in those with IE]. At first TDM assessment, median (min–max) ceftobiprole Css were 15.7 (3.4–27.4) mg/L in patients with BSI and 11.2 (3.1–27.4) mg/L in those with IE, respectively. The aggressive PK/PD target of efficacy of *f*Css/MIC of 4–8 was attained in almost all cases [91.6% of patients having BSI (11/12), median (min–max) *f*Css/MIC of 26.9 (2.9–41.8); 100% of those (11/11) having IE, median (min–max) *f*Css/MIC was 22.4 (5.2–92.1)].

**Figure 2:**
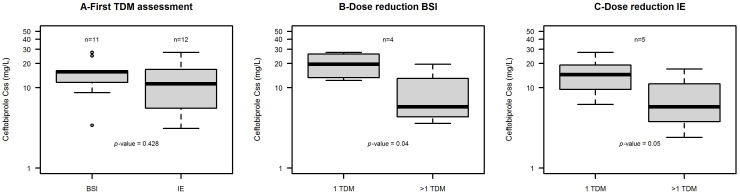
Box and whisker plot of ceftobiprole Css (mg/L). Panel (**A**) shows Css distribution at first TDM assessments in patients with BSI (*n* = 11) vs. patients with IE (*n* = 12). Panel (**B**) shows Css distribution at first TDM assessment vs. after dose reduction in patients with BSI with multiple TDM assessments and dose reduction (*n* = 4). Panel (**C**) shows Css distribution at first TDM assessment vs. after dose reduction in patients with IE with multiple TDM assessments and dose reduction (*n* = 5). The box plot represents the median with 25th–75th percentiles and the whiskers identify the range of normal data. Whiskers are calculated as 25th percentile − (1.5 × IQR) or 75th percentile + (1.5 × IQR), where IQR is the interquartile range. Dots are outliers.

In the vast majority of patients the *f*Css/MIC ratio observed was >10 [90.9% of patients having BSI (10/11) and 83.3% of those having IE (10/12)]. Consequently, in those patients undergoing subsequent TDM reassessments [7/11 of those with BSI (63.6%) and 8/12 of those with IE (66.7%)] ceftobiprole doses were reduced in around half cases [57.1% (4/7) of those with BSI and 62.5% (5/8) of those with IE]. After dosing reduction, the median ceftobiprole Css values declined significantly in both patients with BSI (from 19.6 to 5.8 mg/L, *p* = 0.04) and in those with IE (from 14.5 to 5.8 mg/L, *p* = 0.05). The distribution of ceftobiprole Css at first TDM assessment and after dose reduction for those patients having multiple TDM assessments in both groups is reported in Figure 2.

After dosing reduction, the median *f*Css/MIC ratio values were significantly reduced in both patients with BSI (from 21.84 to 7.35, *p* = 0.013) and in those with IE (from 32.1 to 18.8, *p* = 0.05). Importantly, in all cases the aggressive PK/PD target of efficacy (*f*Css/MIC ratio of 4–8) was still attained [median (min–max) ceftobiprole *f*Css/MIC of 9.2 (5.5–60.1) in patients with BSI and of 21.4 (4.0–126.3) in those with IE]. The attainment of the PK/PD targets for ceftobiprole at first TDM assessment and after dose reduction for those patients having multiple TDM assessment in both groups is reported in Table 3.

Interestingly, dosing increases were never needed, neither for daptomycin nor for ceftobiprole.

Overall, the daptomycin–ceftobiprole combination therapy was well-tolerated. Regarding daptomycin, elevation of creatine phosphokinase (CPK) was observed in only 2/23 cases (8.7%) (one had CPK at 232 UI/L under a daptomycin AUC_24h_ of 1619.1 mg∙h/L; the other had CPK at 173 UI/L under a daptomycin AUC_24h_ of 1138.0 mg∙h/L). In both cases daptomycin dosage reduction was applied and CPK gradually decreased during treatment. Unfortunately, hyper-eosinophilia was a quite frequent occurrence [69.6% of patients (16/23) had at least once detection during treatment (mild in 5/23 patients, moderate in 7/23 patients and severe in 4/23 patients)]. Regarding ceftobiprole, one patient developed a maculopapular rash likely related to this drug. The adverse event resolved rapidly after ceftobiprole discontinuation.

### 2.3. Assessment of Clinical Outcomes

**Figure 3:**
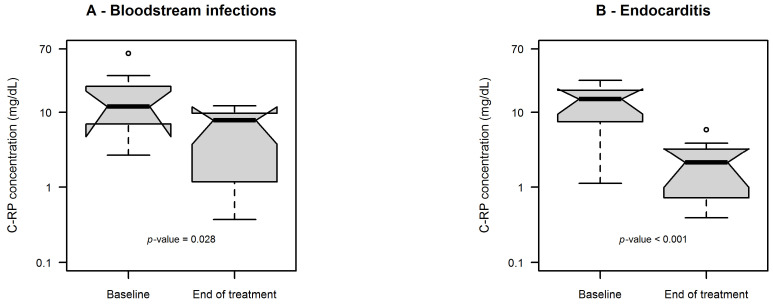
Box and whisker plot of C-RP concentrations at baseline vs. at end of treatment in patients with bloodstream infections (**A**) and endocarditis (**B**). The box plot represents the median with 25th–75th percentiles and the whiskers identify the range of normal data. Whiskers are calculated as 25th percentile − (1.5 × IQR) or 75th percentile + (1.5 × IQR), where IQR is the interquartile range. Dots are outliers.

The distributions of C-reactive protein (C-RP) values at baseline vs. end of treatment in patients with BSI and IE are depicted in Figure 3A and Figure 3B, respectively. The median C-RP values significantly decreased at end of treatment vs. baseline both in patients with BSI (11.9 vs. 7.8 mg/dL, *p* = 0.028) and in those with IE (15.1 vs. 2.1 mg/dL, *p* < 0.001).

Among patients with IE, cardiosurgery was needed in 33.3% of cases (4/12). Microbiological eradication was achieved in all but one patient (in that patient microbiological failure was due to rapid clinical deterioration related to the IE and he passed away). The median time to bloodstream bacterial clearance was 6 days from treatment initiation (IQR 3–9). At the end of the 30-day follow-up, 4 patients died, but only two of these due to the underlying infectious disease (1 with BSI and 1 with IE). One patient with BSI had a relapse 82 days after stopping the treatment, likely due to previous misdiagnosis and inadequate management. Among the 21 evaluable patients, clinical cure was observed in 77.8% of patients with BSI (7/9) and in 91.7% of those with IE (11/12).

## 3. Discussion

This study first performed a PK/PD analysis of high-dose daptomycin in combination with CI ceftobiprole in a case series of documented staphylococcal BSI and/or IE. Overall, the findings may provide novel real-world evidence on the high likelihood of PK/PD target attainment of this strategy for both agents and on the opportunity of applying TDM-guided dosing reduction as a strategy for improving long-term treatment safety whenever clinical isolates are very susceptible to both of the agents.

High-dose daptomycin is still burdened by quite high mortality (20–30%) [3,4,18] and microbiologic failure (10–16%) [19] rates. Persistent bacteremia and mortality remain concerns, particularly in complicated infections or when vancomycin MICs are elevated [3,4,15,16]. As far as combination therapy in targeted treatment of staphylococcal BSI and IE is concerned, the CAMERA-2 trial [14] was the largest randomized clinical trial of β-lactam combination therapy in this setting. In that study, adding an anti-MSSA β-lactam (flucloxacillin, cloxacillin, and cefazolin) to standard vancomycin or daptomycin therapy resulted in more rapid clearance of bacteraemia. However, the potential microbiological benefit did not translate into improved composite clinical endpoints, and the trial was prematurely discontinued because of significantly higher adverse events, particularly acute kidney injury, in the combination therapy arm [14].

Recently, combining daptomycin with a fifth generation anti-MRSA cephalosporin, namely ceftobiprole or ceftaroline, was suggested as a potential valuable approach for properly dealing with MRSA BSI [20]. Interestingly, the findings of a recent pilot randomized study daptomycin plus ceftaroline showing better clinical cure rates and fewer adverse events compared to monotherapy with daptomycin or vancomycin could support this contention [31]. While waiting for the findings of a well-designed randomized clinical trial providing definitive evidence on this issue [20], our study may act as a proof of concept of how this combination therapy strategy may perform in the setting of MRSA/MRSE BSI or IE and could be properly managed by means of a TDM-guided approach.

A key implication of the findings is that PK/PD target attainment was always optimal with high-dose daptomycin plus CI ceftobiprole. However, it should be highlighted that the TDM-guided approach allowed to apply relevant dosing reductions without compromising efficacy whenever documented staphylococcal BSI or IE were caused by very susceptible clinical isolates to both agents. Surveillance studies consistently showed that the MIC distribution of MRSA for daptomycin remains low, with MIC_90_ values ≤ 0.25 mg/L in several settings [2,8,32]. When MICs are low, maintaining maximal daptomycin dosing throughout prolonged treatment courses may result in unnecessary overexposure without incremental microbiological benefit. Moreover, our findings suggest the importance of model-informed precision dosing (MIPD) in the management of staphylococcal BSI and IE. In particular, Bayesian estimation of daptomycin AUC provided a more accurate estimation of drug exposure, thus allowing precise individualization of daptomycin dosing [33]. Specifically, trough concentration showed wide inter-individual variability [34], with the risk that trough-based strategies may misclassify exposure in a substantial proportion of patients [35]. By contrast, the use of MIPD for ceftobiprole appears less compelling due to the fact that its clearance is linearly associated with renal function, thus making drug pharmacokinetics more predictable.

The safety profile observed in our patients reinforces the value of individualized dosing strategies. Higher daptomycin exposure has been associated with an increased probability of CPK elevation [35]. Interestingly, the fact that only two patients had a mild increase in CPK that recovered after dosing adaptation may suggest that this strategy may be valuable in improving drug safety [36]. Although some authors suggested that withholding one daptomycin dose during treatment may allow patients with asymptomatic, elevated CPK concentrations to continue therapy with subsequent CPK level normalization [37], it is undoubtful that the TDM-guided approach may be more effective in dealing with this issue. The findings of hyper-eosinophilia were quite common, consistent with previous reports supporting the need of analyzing eosinophil count when using high daptomycin doses [36]. Previous studies have shown that most cases of daptomycin-associated eosinophilia are asymptomatic and reversible, although rare cases of eosinophilic pneumonia have been described [38,39]. Overall, the present findings highlight the importance of laboratory surveillance of both CPK and absolute eosinophilic count during prolonged targeted therapy and suggest that eosinophilia and CPK elevation, while frequent, are often clinically manageable and do not necessarily preclude continuation of therapy. TDM of daptomycin may be especially valuable to prevent these toxicity risks, whenever daptomycin AUC_24h_ > 939 mg·h/L is associated with C-RP > 21.6 mg/dL, since in these cases the risk of severe adverse events, namely eosinophilic pneumoniae and myotoxicity, may be increased by more than 30-fold and 8-fold, respectively, during long-term treatment [36]. As far as ceftobiprole is concerned, this cephalosporine is generally safe even in settings of frail elderly patients [40]. In a recent retrospective observational study conducted among 249 Spanish patients treated with ceftobiprole (alone or in combination with other antimicrobials) for pneumonia (55.8%), skin and soft tissue infections (21.7%) and BSI (17.7%), adverse events were recorded in only 3.6% (9/249) of patients. In this light, reducing overexposure is not mandatory for preventing toxicity but it could indeed be helpful in reducing unuseful potentially harmful selective pressure. This could may be particularly relevant in geographic areas where high resistance rates to ceftobiprole around 15% have been reported, namely Italy [41] or sub-Saharan Africa [42].

We recognize that our study has some limitations. The monocentric design, the limited sample size, and the absence of a comparator group may limit the generalizability of our findings and preclude us from establishing the real advantage of this combination therapy vs. monotherapy. The high prevalence of hyper-eosinophilia, although being asymptomatic, may warrant cautious interpretation in terms of long-term safety. Finally, our dose reduction strategy should be externally validated in clinical context having different patterns of pathogen susceptibility. Nonetheless, the detailed PK/PD analysis and the focus on targeted treatment decision-making may be points of strength in our study.

## 4. Materials and Methods

### 4.1. Study Design and Clinical Setting

This retrospective monocentric observational study was carried out between January 2024 and December 2025 at the IRCCS, Azienda Ospedaliero-Universitaria di Bologna, Italy, among patients receiving a TDM-guided combination therapy with high-dose daptomycin and CI ceftobiprole for treating documented staphylococcal BSI or IE.

Bloodstream infection by *Staphylococcus aureus* was defined as the isolation of this species from at least one blood culture. BSI by *Staphylococcus epidermidis* was defined as the isolation of this microorganism from two or more separate blood cultures in the presence of clinical signs and symptoms consistent with infection [10]. BSI sources were established according to US Centers for Disease Control and Prevention (CDC) criteria [43]. BSI was defined as “primary” in case of unidentified source of infection.

The diagnosis of IE was established according to the latest modified 2023 Duke-ISCVID Criteria [44]. Specifically, the Duke criteria were assessed in all patients presenting risk factors for IE (i.e., prosthetic heart valves, congenital heart disease, intracardiac devices, intravenous drug use, etc.). IE cases were classified as involving either prosthetic or native valves based on the patient’s characteristics. Transthoracic and/or trans-esophageal echocardiography was performed as the first diagnostic step after blood culture positivity. In patients with prosthetic valves, if echocardiography was negative, FDG-PET/CT scan was subsequently performed to confirm or exclude the diagnosis of IE [44]. Only patients with definite or possible IE were included in this study [44]. Patients with BSI and/or IE were managed according to current international guidelines, most recent available evidence and clinical judgment [11,45]. At our center, all patients with a diagnosis of BSI and/or IE are treated with targeted antimicrobial combination therapy based only on daptomycin plus continuous infusion (CI) ceftobiprole until end of treatment or oral step-down therapy. No patient received previous antibiotic treatment before diagnosis.

Treatment was started with standard dosing regimens: daptomycin at a dose of 8–10 mg/kg every 24 or 48 h in patients having eGFR ≥ 30 or <30 mL/min/1.73 m^2^, respectively; ceftobiprole with a loading dose of 500 mg over 2 h followed by a maintenance dose of 1500 mg/daily by CI in patients with eGFR > 50 mL/min/1.73 m^2^, 1000 mg/daily by CI if eGFR 30–50 mL/min/1.73 m^2^, and 500 mg/daily by CI if eGFR < 30 mL/min/1.73 m^2^.

At our tertiary-care hospital TDM is routinely performed from Monday to Friday as part of a program of antimicrobial stewardship aimed at optimizing drug exposure and minimizing the risk of antimicrobial resistance [46]. Patients underwent first TDM assessment of both daptomycin and ceftobiprole after at least 48–72 h from starting therapy, and subsequently every 48–72 h whenever feasible. TDM of daptomycin was performed by assessing both the trough concentrations, namely 5–15 min before the scheduled dose, and the peak concentrations, namely at the end of a 5 min infusion. TDM of ceftobiprole was performed by assessing steady-state plasma concentration (Css).

Plasma daptomycin concentrations were measured by means of a liquid chromatography-tandem mass spectrometry (LC/MS-MS) commercially available method (Recipe Chemicals + Instruments GmbH, Munich, Germany), with a lower limit of quantification of 1.5 mg/L. Plasma ceftobiprole concentrations were measured by LC/MS-MS analytic method as previously described [42]. The lower limit of quantification was 0.5 mg/L.

At each TDM assessment, daptomycin AUC_24h_ was estimated by means of a Bayesian software (MwPharm++, version 2.4.0.363, Mediware, Prague, Czech Republic) and the PK/PD target of efficacy was set at both AUC_24h_/MIC ≥ 666 [47] and AUC_24h_/MIC ≥ 1081 [48]. Specifically, the lowest value was the target that has been associated with a 1-log kill of *S. aureus* in an early neutropenic murine thigh infection model [47]. The highest value was the target that has been associated with a good probability of clinical success in an exposure-response study including 78 adult patients with staphylococcal bacteremia with or without endocarditis [48]. Both targets were previously adopted in other studies assessing daptomycin efficacy in patients having endovascular infections [48,49]. An absolute daptomycin AUC_24h_ value > 939 mg∙h/L was set as potential threshold of drug-related toxicity, namely myopathy and hyper-eosinophilia. This was the cut-off value that has been identified by means of log-rank test in a large cohort of patients (*n* = 1130) receiving treatment with high-dose daptomycin for bone and joint infections [36].

PK/PD target of efficacy for ceftobiprole was set at an aggressive PK/PD target defined as a free Css to MIC ratio (*f*Css/MIC) of 4–8 (corresponding to a 100% T > 4–8 × MIC) [29]. A *f*Css/MIC ratio > 10 was set as value for ceftobiprole dosing reduction, as previously reported for other beta-lactams [50]. Total ceftobiprole concentrations were measured and the *f*Css was calculated based on a plasma protein binding of 16% [51]. Table 4 summarizes the clinical pharmacological thresholds set for assessing efficacy and safety of both daptomycin and ceftobiprole.

Microbiological susceptibility of *Staphylococci* to both daptomycin and ceftobiprole was tested by broth microdilution.

### 4.2. Data Collection

Demographic (age, gender, weight, and height) and clinical (Charlson Comorbidity index, SOFA Score at admission, type and site of infection) were collected at starting treatment. Microbiological (type of bacterial isolates together with daptomycin and ceftobiprole antimicrobial susceptibility testing), pharmacological data (daptomycin and ceftobiprole starting dose, frequency and entity of dose adjustments, treatment duration and TDM values, switch to oral therapy) and laboratory parameters (such as serum albumin, alanine aminotransferase, aspartate aminotransferase, CPK, absolute eosinophil count and C-RP) were collected during treatment. Serum creatinine was assessed at each TDM session. The estimated glomerular filtration rate was calculated by means of the CKD-EPI formula [52].

### 4.3. Clinical Evaluation and Safety

Clinical outcome was assessed at the end of treatment and at 7, 14 and 30 days follow-up. Clinical cure was defined as a composite of resolution of all signs and symptoms of infection coupled with defervescence, microbiological eradication, C-RP reduction or bloodstream bacterial clearance vs. baseline. Microbiological eradication was defined as sustained negative follow-up blood cultures. Follow-up blood cultures were drawn between 48 h and 7 days after the index blood culture. Results of follow-up blood cultures were classified as positive for the same pathogen or negative.

Treatment inefficacy was defined in presence of infection relapse or death caused by worsening of the infection-related clinical conditions. Relapse was defined as a new episode of IE and/or BSI caused by the same microorganism occurring after completing antibiotic therapy. Breakthrough infection was defined as the isolation of the same microorganism during ongoing, appropriate antibiotic treatment. Duration of antibiotic treatment was defined as the number of consecutive days of combination antimicrobial therapy. Time to blood culture clearance was defined as the number of days from the index blood culture from starting antibiotic therapy to the first documented negative follow-up blood culture.

Safety of daptomycin therapy was assessed by evaluating CPK and absolute eosinophil count during treatment. A CPK value > 170 UI/L or an absolute eosinophil count > 0.5 × 10^9^/L (mild eosinophilia 0.5–1.5 × 10^9^/L, moderate eosinophilia 1.5–5.0 × 10^9^/L, severe eosinophilia > 5.0 × 10^9^/L) were considered signs of potential daptomycin toxicity [53].

### 4.4. Statistical Analysis

The Kolmogorov–Smirnov test was used to assess whether data were normally or non-normally distributed. Accordingly, means ± SD or medians and IQR were used for descriptive statistics. The statistical difference between groups was assessed by means of Student *t*-test or Mann–Whitney U test, as appropriate. All statistical analyses and graphs were performed with R version 4.3.3 (The R Foundation for Statistical Computing, Vienna, Austria).

## 5. Conclusions

In conclusion, this study provides the first real-life proof of concept of the pharmacological performance and tolerability of high-dose daptomycin plus CI ceftobiprole as a targeted treatment strategy for staphylococcal BSI and IE. The findings underscore the value of integrating PK/PD principles, MIC-informed dosing, and MIPD for optimizing the balance between efficacy and toxicity by minimizing unnecessary overexposure. Larger multicenter studies are warranted to further define the role of this combination therapy and to refine precision dosing strategies in severe staphylococcal BSI/IE.

## Figures and Tables

**Table 1:** Demographic and clinical characteristics.

Variable	BSI (*n* = 11)	IE (*n* = 12)
Age (years)	72 (63–81)	74 (61–82)
Gender (male)	5 (45)	9 (75)
Weight (kg)	75 (60–84)	68.5 (60–74)
BMI (kg/m^2^)	27.3 (22.7–29.9)	22.2 (21.7–25.2)
AST (IU/L)	17.0 (14.8–25.5)	23.0 (17.8–32.5)
ALT (IU/L)	15.0 (9.0–24.3)	20.0 (13.3–32.8)
Serum albumin (g/dL)	2.8 (2.7–3.5)	3.1 (2.7–3.5)
Serum creatinine (mg/dL)	0.9 (0.8–1.2)	0.9 (0.8–1.6)
eGFR (mL/min/1.73 m^2^)	69.0 (61.5–83.8)	70.0 (42.0–92.0)
Charlson Comorbidity Index	6 (5–8)	5 (3–8)
SOFA score at diagnosis	4 (3–6)	2 (1–4)
Septic shock	0 (0.0)	2 (18.2)
Ward of admission		
Medicine	6 (54.5)	7 (58.3)
Surgery	0 (0.0)	3 (25.0)
ICU	5 (45.5)	2 (16.7)
Microbiological isolates		
MRSA	7 (63.6)	10 (83.4)
MSSA	2 (18.2)	0 (0.0)
MRSE	2 (18.2)	2 (16.6)
Pharmacological treatment		
Daptomycin dose (mg/kg daily) *	9.1 (8.3–9.5)	6.0 (4.3–9.7)
Daptomycin AUC_24h_ (mgh/L) *	1294.7 (846.5–1386.8)	743.4 (623.0–1002.6)
Ceftobiprole dose (g/daily by CI) *	1000 (750–1500)	1250 (500–1500)
Ceftobiprole Css (mg/L) *	14.4 (8.2–17.1)	10.1 (5.6–15.4)
Duration of combination therapy (days)	13 (4–15)	21 (18–26)
Switch to oral/long-acting therapy	1 (9.1)	6 (50.0)
Clinical outcome		
Clinical cure	7/9 (77.8)	11/12 (91.7)
Death for infection	1 (9.1)	1 (8.3)
Relapse of infection	1 (9.1)	0 (0.0)
Dead for other reasons	2 (18.2)	0 (0.0)

ALT, alanine aminotransferase; AUC_24h_, 24 h-area under the curve; Css, steady-state concentration; AST, aspartate aminotransferase; BMI, body mass index; BSI, bloodstream infection; eGFR, estimated glomerular filtration rate; ICU, intensive care unit; IE, infective endocarditis; MRSA, methicillin-resistant *Staphylococcus aureus*; MRSE, methicillin-resistant *Staphylococcus epidermidis*; MSSA, methicillin-susceptible *Staphylococcus aureus.* Data are expressed as median (IQR) or as number (%). * data are referring to the whole treatment duration.

**Table 2:** Proportion of patients with BSI and IE who attained the PK/PD efficacy targets of daptomycin (AUC_24h_/MIC ≥ 666 and AUC_24h_/MIC ≥ 1081) at first and subsequent TDM assessments.

PK/PD Parameter	BSI		IE	
	First TDM Assessment (*n* = 11)	Subsequent TDM Assessments (*n* = 5)	First TDM Assessment (*n* = 12)	Subsequent TDM Assessments (*n* = 9)
AUC_24h_/MIC ≥ 666	11 (100%)	5 (100%)	12 (100%)	9 (100%)
AUC_24h_/MIC ≥ 1081	11 (100%)	5 (100%)	12 (100%)	9 (100%)

AUC_24h_, 24 h-area under the curve; BSI, bloodstream infection; IE, infective endocarditis; MIC, minimum inhibitory concentration (mg/L).

**Table 3:** Proportion of patients with BSI and IE who attained the PK/PD efficacy target of ceftobiprole (*f*Css/MIC 4–8) at first and subsequent TDM assessments.

PK/PD Parameter	BSI		IE	
	First TDM Assessment (*n* = 11)	Subsequent TDM Assessments (*n* = 7)	First TDM Assessment (*n* = 12)	Subsequent TDM Assessments (*n* = 8)
*f*Css/MIC 4–8	10 (90.1%)	7 (100%)	12 (100%)	8 (100%)

BSI, bloodstream infection; Css, steady-state plasma concentration (mg/L); IE, infective endocarditis; MIC, minimum inhibitory concentration (mg/L).

**Table 4:** Clinical pharmacological parameters used for evaluating efficacy and toxicity for both daptomycin and ceftobiprole.

Antimicrobial Agent	Efficacy	Safety
Daptomycin	AUC_24h_/MIC > 666 and AUC_24h_/MIC > 1081	AUC_24h_ > 939 mg·h/L
Ceftobiprole	*f*Css/MIC 4–8	*f*Css/MIC > 10

AUC_24h_, 24 h-area under the curve; *f*Css free steady-state concentration; MIC, minimum inhibitory concentration.

## Data Availability

The data presented in this study are available upon request from the corresponding author. The data are not publicly available due to privacy concerns. The de-identified individual participant data that underlie the results reported in this article (including text, tables, and figures) will be made available together with the research protocol for non-commercial, academic purposes. Additional supporting documents may be available on request.
